# Signal transducer and activator of transcription 3 (STAT3) controls susceptibility to Epstein-Barr virus reactivation in B cells

**DOI:** 10.1186/1750-9378-7-S1-P42

**Published:** 2012-04-19

**Authors:** Erik R Hill, Shane C McAllister, Stuart Tangye, Sumita Bhaduri-McIntosh

**Affiliations:** 1 Department of Pediatrics, Stony Brook University, and Children’s Hospital at Stony Brook, Stony Brook, NY, USA; 2 Immunology Program, Garvan Institute of Medical Research, Sydney, Australia; 3 Department of Molecular Genetics and Microbiology, Stony Brook University, Stony Brook, NY, USA

## 

Epstein-Barr Virus (EBV) is known to cause malignancies in immunocompromised individuals, including lymphomas and lymphoproliferative disorders. Reactivation of EBV from latency is important in the pathogenesis of such malignancies. Therapeutically, oncolysis of tumors harboring lytic EBV holds promise. However, exposure of latently infected cultured B cells to lytic cycle inducing stimuli results in virus reactivation within only 20% to 50% of cells. Host cell determinants, that govern this susceptibility to lytic reactivation within latently infected B cells, are not well understood. Our research has previously identified that higher levels of signal transducer and activator of transcription 3 (STAT3) within cells correlate with resistance to EBV lytic reactivation. We now investigate whether inhibiting function of STAT3 promotes susceptibility of latently infected B cells to known lytic cycle inducing stimuli or results in induction of EBV lytic cycle. We found that pharmacological inhibition of STAT3 phosphorylation by Janus Kinase 2 (JAK2) inhibitors (AG490 or WP1066) in latently infected cells (HH514-16 Burkitt lymphoma and B95-8 lymphoblastoid cells) increased lytic reactivation by chemical stimuli that function by distinct mechanisms. Moreover, functional inhibition of STAT3 alone resulted in lytic reactivation in these cells. EBV-Lymphoblastoid cell lines (LCL) newly generated from healthy individuals also demonstrated increased susceptibility to lytic cycle inducing stimuli in the presence of AG490 but variable susceptibility to lytic reactivation when exposed to AG490 alone. Since this lack of uniform response to AG490 alone could be due to inadequate functional suppression of STAT3, we examined EBV-LCLs derived from patients with Autosomal Dominant Hyperimmunoglobulin-E syndrome (AD-HIES or Job syndrome). Patients with AD-HIES carry a dominant negative mutation in their *Stat3* gene resulting in lower basal levels of functional STAT3. When LCLs from AD-HIES patients were exposed to a JAK2 inhibitor alone, we observed a strong increase in lytic reactivation by expression of early and late lytic antigens over LCLs derived from healthy individuals. Lytic reactivation in the presence of AG490 occurred in these cells despite lack of discernable increase from basal levels of expression of ZEBRA, the viral lytic switch protein, when compared to cells not exposed to AG490. Thus, STAT3 is important in determining susceptibility to EBV reactivation. Fully understanding how STAT3 governs such susceptibility can lead to novel therapeutic strategies for EBV-related diseases.

